# Draft Genome Sequence of an Atypical Highly Virulent Rabbit *Staphylococcus aureus* Strain

**DOI:** 10.1128/genomeA.01049-17

**Published:** 2017-10-19

**Authors:** Zoltán Német, Ervin Albert, Tibor Nagy, Ferenc Olasz, Endre Barta, János Kiss, Ádám Dán, Krisztián Bányai, Katleen Hermans, Imre Biksi

**Affiliations:** a Department and Clinic for Production Animals, University of Veterinary Medicine Budapest, Üllő, Hungary; b Agricultural Biotechnology Institute of the National Agricultural Research and Innovation Centre, Gödöllő, Hungary; c Veterinary Diagnostic Directorate, National Food Chain Safety Office (NFCSO), Budapest, Hungary; d Institute for Veterinary Medical Research, Hungarian Academy of Sciences, Budapest, Hungary; e Department of Pathology, Bacteriology and Avian Diseases, Ghent University, Ghent, Belgium

## Abstract

Rabbit staphylococcosis is one of the most important diseases in industrial rabbit production. We report here the draft genome sequence of *Staphylococcus aureus* strain 380/11, an atypical highly virulent (aHV) rabbit *Staphylococcus aureus* strain.

## GENOME ANNOUNCEMENT

Staphylococcosis has a major economic impact on rabbit farming. Infections caused by virulent strains result in severe, often fatal diseases (1).

An isolate originating from a subcutaneous abscess of a fattener rabbit, with a phenotype typical of Hungarian commercial rabbit farm *Staphylococcus aureus* strains, identified as 380/11, was used for whole-genome sequencing (WGS). This strain shows the multiplex PCR pattern specific for aHV *S. aureus* strains (2). This genotype was rarely isolated from diseased rabbits (3) but currently is the most prevalent genotype at Hungarian commercial rabbit farms (4).

Total DNA was subjected to 2 × 300-bp paired-end Illumina MiSeq sequencing at the Department of Biochemistry, Faculty of Medicine, University of Szeged, Hungary. A total of 3.64 million read pairs were recorded, and the estimated coverage of the whole genome is 700×.

The estimated coverage of the subsets of reads was adjusted to 30×, and reads were assembled *de novo* using MIRA version 4.0.2 (5), A5 pipeline version 20130326 (6), and SeqMan NGen version 4.1.2 [25] (DNAStar version 10). Scaffolds were built from different assemblies using Mauve version 2.3.1 (7) and a Geneious version 8.1.2 (8) plug-in. This resulted in a total of 15 scaffolds containing 2,631,087 nucleotides. The average G+C content is 32.7%.

Scaffolds were submitted to the Rapid Annotations using Subsystems Technology (RAST) annotation server (9). The taxon was set to “*Staphylococcus aureus*” (1280.2034), the genetic code to “11 (Archaea, Bacteria),” the annotation scheme to “ClassicRAST,” and “preserve gene calls,” “automatically fix errors,” “fix frameshifts,” and “backfill gaps” to “no.” We have obtained 2,567 annotated genes, 52 tRNAs, and 11 rRNAs. The total coding sequences (CDS) comprise 2,295,057 bases, which is 87.22% of all nucleotides.

A comparative analysis of virulence genes will be conducted in order to evaluate the relationships between typical and atypical highly virulent rabbit *Staphylococcus aureus* strains.

### Accession number(s).

This whole-genome shotgun project has been deposited at DDBJ/EMBL/GenBank under the accession number LYXH00000000.
